# Treatment of multiple-beam X-ray diffraction in energy-dependent measurements

**DOI:** 10.1107/S1600577523009670

**Published:** 2024-01-01

**Authors:** Melanie Nentwich, Matthias Zschornak, Tina Weigel, Thomas Köhler, Dmitri Novikov, Dirk C. Meyer, Carsten Richter

**Affiliations:** a Deutsches Elektronen-Synchrotron DESY, Notkestraße 85, 22607 Hamburg, Germany; b Institute of Experimental Physics, Technical University Bergakademie Freiberg, 09596 Freiberg, Germany; c Leibniz-Institut für Kristallzüchtung, Max-Born-Straße 2, 12489 Berlin, Germany; ESRF and Université Grenoble Alpes, France

**Keywords:** resonant elastic X-ray scattering, multiple-beam X-ray diffraction, Renninger effect, data processing

## Abstract

During X-ray diffraction experiments on single crystals, the diffracted beam intensities may be affected by multiple-beam X-ray diffraction (MBD). This effect is particularly frequent at higher X-ray energies and for larger unit cells. The appearance of this so-called Renninger effect often impairs the interpretation of diffracted intensities. This applies in particular to energy spectra analysed in resonant experiments, since during scans of the incident photon energy these conditions are necessarily met for specific X-ray energies. This effect can be addressed by carefully avoiding multiple-beam reflection conditions at a given X-ray energy and a given position in reciprocal space. However, areas which are (nearly) free of MBD are not always available. This article presents a universal concept of data acquisition and post-processing for resonant X-ray diffraction experiments. Our concept facilitates the reliable determination of kinematic (MBD-free) resonant diffraction intensities even at relatively high energies which, in turn, enables the study of higher absorption edges. This way, the applicability of resonant diffraction, *e.g.* to reveal the local atomic and electronic structure or chemical environment, is extended for a vast majority of crystalline materials. The potential of this approach compared with conventional data reduction is demonstrated by the measurements of the Ta *L*_3_ edge of well studied lithium tantalate LiTaO_3_.

## Abstract 2

An approach to reliably extract the desired intensities and filter out multiple-beam X-ray diffraction, which often causes interference for high X-ray energies and for large unit cells, is presented. Here, a universal concept of data acquisition and post-processing for resonant X-ray diffraction experiments is described, including the measurement of the energy-dependent intensity at several azimuth angles and subsequently only considering the unaffected data points.

## 1. Introduction

X-ray diffraction (XRD) from perfect single crystals is described by the dynamical theory of diffraction (Authier, 2005 ▸; Batterman & Cole, 1964 ▸). This theory also describes the interference effects that appear when the Bragg condition is fulfilled for several reflections. This so-called multiple-beam X-ray diffraction (MBD) may result in enhancement or dampening of diffracted intensity (Renninger, 1937 ▸; Newville, 2021 ▸; Kohn, 1979 ▸; Kohn & Kazimirov, 2012 ▸; Besirganyan *et al.*, 1984 ▸). In contrast, diffraction from slightly imperfect or small crystals can be described by the kinematic theory of diffraction as a small lattice coherence length prevents the multiple wave interference effect to a large extent (Holý *et al.*, 1994 ▸; Krivoglaz, 1996 ▸; Juretschke, 1984 ▸). Most practical applications assume the kinematic approach to be valid and dynamical effects are treated as a source of experimental error. However, especially for hard X-rays, large unit cells, and weak or even ‘forbidden’ reflections, the measured intensities can be dominated by MBD (Gabrielyan & Kohn, 1981 ▸), also called the Renninger effect (Newville, 2021 ▸) or *Umweganregung*. The investigation of the fine structure oscillations in resonant elastic X-ray spectroscopy (REXS) experiments (Richter *et al.*, 2018 ▸; Weigel *et al.*, 2023 ▸; Nentwich *et al.*, 2016 ▸) suffers particularly from these effects (see Section 2), which have been either overlooked or perceived as disruptive and hence avoided (Laligant *et al.*, 1989 ▸; Baruchel, 1993 ▸; Massa, 2007 ▸; Petcov, 1989 ▸). Lately, increasing concomitant interpretation facilitates additional information about the material (Hayashi *et al.*, 1999 ▸; dos Santos *et al.*, 2019 ▸; Borcha *et al.*, 2017 ▸; Mikula *et al.*, 2021 ▸; Freitas *et al.*, 2007 ▸; Chang, 2004 ▸, 1982 ▸; Weckert & Hümmer, 1997 ▸). So-called Renninger scans, describing a rotation about the azimuth angle ψ (about the normal of the diffracting lattice planes), reflect the symmetry of the crystal structure and bear information about the orientation of local electronic orbitals in REXS experiments (Zschornak *et al.*, 2014 ▸). Still, the undesired appearance of MBD needs to be carefully handled to obtain clean values of the structure amplitude for data evaluation. One approach is simply to avoid the constellations where this effect occurs, but, as we will show, this is rarely possible. Thus, here we present an approach to correct for MBD in a readily automatable way.

Recently, Kozlovskaya *et al.* (2021 ▸) presented an approach to avoid MBD *a priori*. Prior to the measurements, they calculated so-called Renninger maps displaying the intensity depending on both X-ray energy and azimuth angle ψ (Weckert & Hümmer, 1997 ▸; Dmitrienko, 2009 ▸; Ovchinnikova *et al.*, 2020 ▸; Richter, 2021 ▸; Walz, 2011 ▸). Based on these maps, they determined the sample orientations for which the desired energy scan of TeO_2_ at approximately 4.94 keV (Te *L*_1_ edge) was free of MBD.

**Figure 1:**
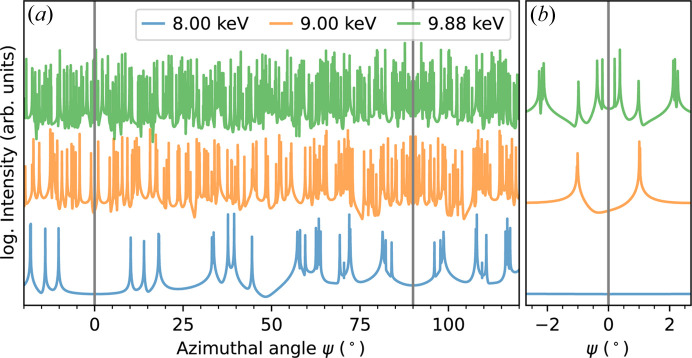
(*a*) Simulation of MBD during an azimuthal rotation at different fixed energies demonstrated here for the ‘forbidden’ 0 3 15 reflection of stoichiometric LiTaO_3_. (*b*) Detailed view of the MBD in the high-symmetric position at ψ = 0°. The addition of an intensity offset allows better visualization.

However elegant this approach is, it may not always be applicable. Especially for large ratios of unit-cell dimensions to wavelength, the number of reflections close to the Ewald sphere may become too high (Baruchel, 1993 ▸). Given a compound of interest, the cell dimensions are fixed and the photon energy alone determines this density. As an example, Fig. 1 ▸ shows the influence of the energy on the calculated azimuthal dependence of the ‘forbidden’ 0 3 15 reflection intensity of ferroelectric lithium tantalate LiTaO_3_. Scans of ψ reflect the symmetry of the crystal structure and bear information about the orientation of local electronic orbitals in REXS experiments (Zschornak *et al.*, 2014 ▸), as highlighted by the grey lines in Fig. 1 ▸. Additionally, this figure shows that, although the LiTaO_3_ unit cell is still rather small and X-ray energies of 9.88 keV (Ta *L*_3_ edge) are moderate, hardly any azimuthal range remains unaffected by MBD.

Fig. 1 ▸ is based on the structure solution of ICSD 9537 (Abrahams & Bernstein, 1967 ▸) and on the equations given by Weckert & Hümmer (1997 ▸) implemented in the Python module *pyasf* (Richter, 2021 ▸). Trigonal lithium tantalate is a well studied material with regards to structure (Abrahams & Bernstein, 1967 ▸), electrical and chemical properties (Smith & Welsh, 1971 ▸; Köhler *et al.*, 2016 ▸, 2021*a* ▸; de Vivanco *et al.*, 2020 ▸), growth (Barns & Carruthers, 1970 ▸; Miyazawa & Iwasaki, 1971 ▸; Brandle & Miller, 1974 ▸; Furukawa *et al.*, 1999 ▸), and defects (Vyalikh *et al.*, 2018 ▸; Zotov *et al.*, 1994 ▸; Köhler *et al.*, 2021*b* ▸, 2023 ▸), and serves as a model material here.

Evidently, the approach of *a priori* calculations to avoid the occurrence of MBD in the measurements is limited in its application when the Ewald sphere becomes large. In this work, we present an alternative approach to obtain resonant diffraction spectra free from the Renninger effect, which facilitates the investigation of a larger group of materials even at higher energy absorption edges. In Section 3, we describe the concept of data acquisition and post-processing of REXS data, and in Section 4 we demonstrate the application of this approach to the aforementioned measurements of LiTaO_3_.

## 2. Origin and theory of multiple-beam X-ray diffraction

**Figure 2:**
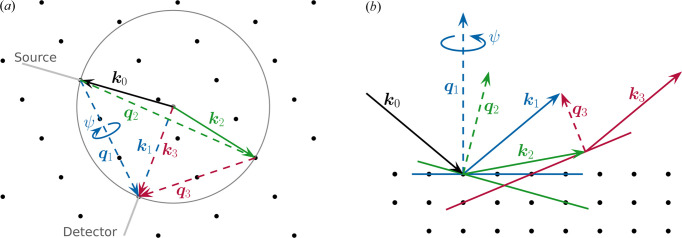
Principle mechanisms for the appearance of the Renninger effect. (*a*) Ewald sphere construction. In addition to the regular Ewald sphere, a third reciprocal lattice point intersects the sphere, indicating an additional diffraction path along reflections **q**_2_ and **q**_3_ instead of only **q**_1_. (*b*) Real space schematic. The primary beam **k**_0_ is diffracted at the blue crystal plane, resulting in the outgoing beam **k**_1_ corresponding to the reflection **q**_1_. At the same time, **k**_0_ is also diffracted at the green and red plane. The resulting beams **k**_2_ and **k**_3_ correspond to the reflections **q**_2_ and **q**_3_, respectively. **k**_1_ and **k**_3_ have the same direction as well as energy, thus the intensity of reflection **q**_1_ and multiple diffracted reflection **q**_3_ are superimposed in a constructive or destructive way.

MBD was first described by Renninger (1937 ▸) when he measured significant intensity at the position of the ‘forbidden’ 2 2 2 reflection of diamond as it was superimposed by MBD. The effect occurs if two reflections are excited simultaneously, *i.e.* when two reciprocal lattice points lie on the Ewald sphere (Newville, 2021 ▸) under consideration of the excitation error (Bethe, 1928 ▸; Weckert & Hümmer, 1997 ▸), see Fig. 2 ▸(*a*). Fig. 2 ▸(*b*) visualizes the effect in real space: the diffracted beam **k**_1_ is the conventional Bragg reflection of the primary beam **k**_0_, caused by diffraction from the blue planes. Simultaneously, **k**_0_ is diffracted in a coherent process at another set of crystallographic planes (green) towards **k**_2_, which now serves as the incoming beam for the red planes, resulting in the detour excited beam **k**_3_. In the rare case that **k**_3_ points in the same direction as **k**_1_, the intensity is either enhanced or damped compared with the signal of **k**_1_ alone, depending on the interaction between those beams being constructive or destructive (dos Santos *et al.*, 2019 ▸). The conditions to observe the Renninger effect are related to the Bragg condition of having a second reflection on the Ewald sphere (*i.e.* highly sensitive to lattice parameters, energy and angular position of the sample). For avoiding or studying the Renninger effect, degrees of freedom are the azimuthal rotation (about angle ψ) of the primary reflection of theX-rayenergy.

The azimuthal rotation corresponds to a rotation of the reciprocal lattice around **q**_1_ in the Ewald sphere, see Fig. 2 ▸(*a*). Depending on the inclination of **q**_2_ with respect to **q**_1_, small rotations may cause additional reciprocal lattice points to leave or enter the surface of this sphere and, thus, violate or fulfil the Laue condition of the MBD. Therefore, the MBD exhibits a larger or a similar angular width compared with other Bragg reflections. The density of reciprocal lattice points in the vicinity of the surface of the Ewald sphere in Fig. 2 ▸(*a*) and, thus, the probability to encounter MBD increases with the ratio of unit-cell dimensions to wavelength (Richter *et al.*, 2014 ▸).

The wave resulting from MBD is the superposition of the primary beam wave **q**_1_ and the wave that is simultaneously diffracted at the lattice planes **q**_2_ and **q**_3_, with **q**_3_ = **q**_1_ − **q**_2_, denoted by their corresponding reciprocal lattice vectors. In general, the interference of two waves is described as their complex sum. In the present case, the formula is based on the structure factors (*F*) involved, corrected for the X-ray scattering strength Γ (Juretschke, 1984 ▸) and for the beam polarization by the geometrical coupling factors α_*nm*_ (Weckert & Hümmer, 1997 ▸). The term of the detour excited wave is the product of both partial waves and a resonance term *R*(**q**_2_) (Weckert & Hümmer, 1997 ▸). In total, the effective structure factor can be calculated by

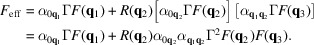

This formula is realized within the Python module *pyasf* (Richter, 2021 ▸), which was used for the calculations presented within this article.

## 3. Data acquisition and processing

**Figure 3:**
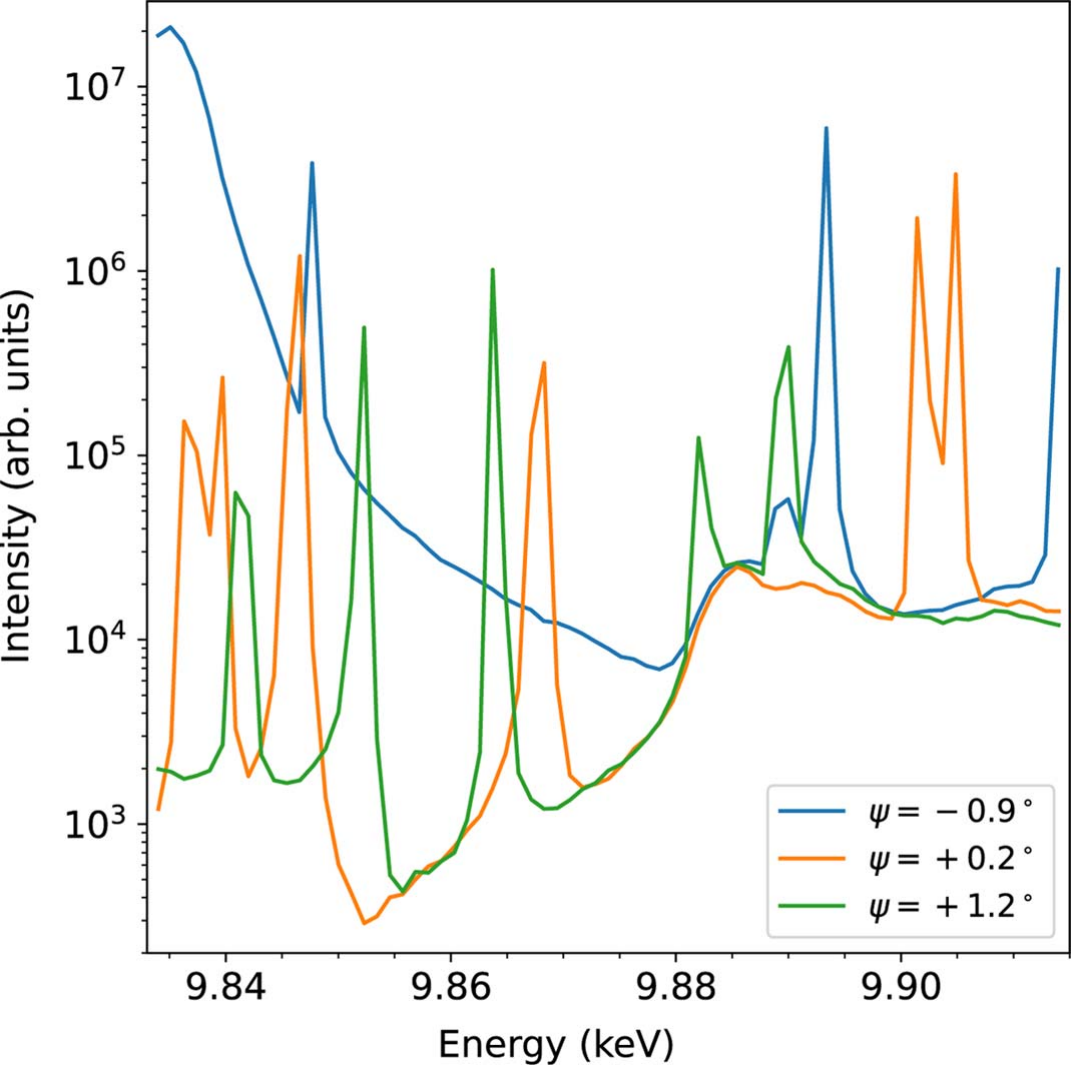
Experimental energy scans at different azimuth angles for the ‘forbidden’ 0 3 15 reflection of congruent LiTaO_3_ at the Ta *L*_3_ edge.

The occurrence of the Renninger effect is unavoidable when recording REX spectra as scanning a range of incident photon energies is usually required. One way to filter out the desired energy dependencies of the two-beam case (in the absence of MBD) is to acquire several scans for the same energy range at arbitrarily chosen azimuth angles, as outlined in Fig. 3 ▸. However, the number of required azimuthal positions to obtain a clean spectrum also depends on the choice of these angles. In this case, due to a high number of reciprocal lattice points close to the Ewald sphere surface, the number of scans was not sufficient. The data were recorded in Bragg geometry with the six-circle Huber diffractometer of beamline P23 at PETRA III synchrotron, Hamburg. The energy resolution of the Si(111) double-crystal monochromator used is 1.3 × 10^−4^ and the divergence is 7.1 µrad × 2.1 µrad. Both the divergence and its energy bandwidth influence the visibility and sharpness of MBD during energy scans. With its beam parameters, beamline P23 represents a typical case.

**Figure 4:**
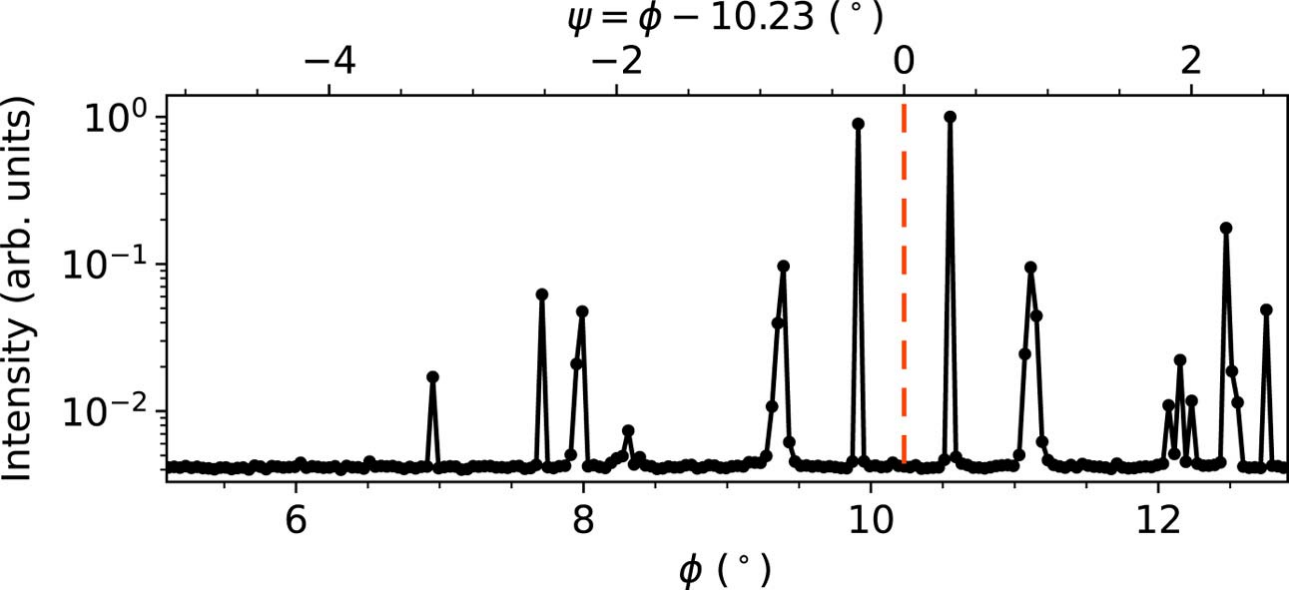
Experimental azimuthal scan of congruent LiTaO_3_ at the Ta *L*_3_ edge for the ‘forbidden’ 0 3 15 reflection realized by rotating ψ/ϕ. The high-symmetry position at ϕ = 10.23° marks the zero position of the azimuth ψ with a red line.

We were able to improve the quality of the spectra by significantly increasing the data redundancy using a larger number of azimuthal positions (angle ψ, ∼25 steps, inner loop) at the expense of a rougher sampling of the photon energy (∼75 steps) in the outer loop. In general, the rotation about the azimuth angle ψ of a given reflection requires a combined rotation of about three (*e.g.* Eulerian) axes of the diffractometer unless the diffracting lattice planes can be aligned perpendicular to one of the rotation axes. At optimized beamlines, continuous coupled scans of several axes are nowadays available, allowing the user to acquire a much higher number of azimuthal data points per unit time. Using a 2D detector allows the separation of the background from the diffracted signal including MBD. The zero position of the azimuthal needs to be defined [*e.g.* following the convention of Schwarzenbach & Flack (1989 ▸)] and determined for the respective crystal under study as demonstrated in Fig. 4 ▸. Here, the measurement settings were such that the inner rotation axis ϕ was nearly parallel to the ψ axis and the high-symmetry position at ϕ = 10.23° was found to be the zero position of ψ.

**Figure 5:**
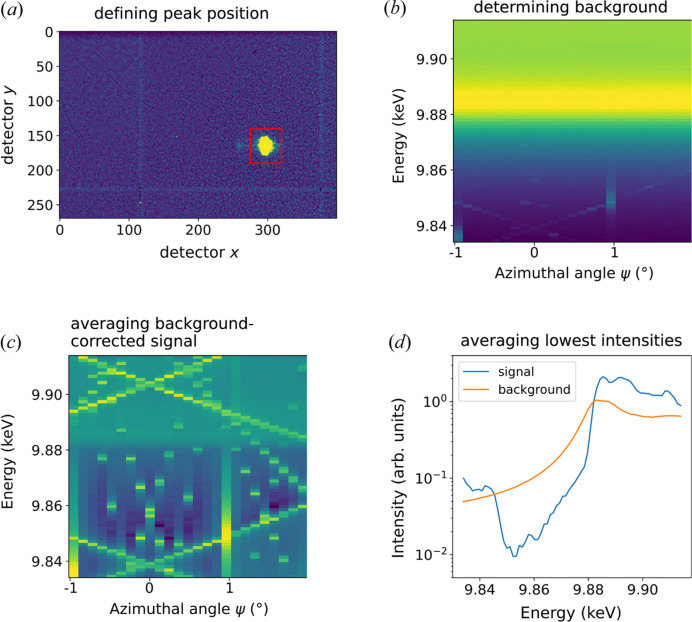
Stepwise procedure to filter out the Renninger effect and background leading to the corrected REXS signal. This is shown for the ‘forbidden’ 0 3 15 reflection near the Ta *L*_3_ edge of congruent LiTaO_3_ from experimental data. The steps include (*a*) manually defining the peak position on the detector frame; (*b*) determining the background intensity for each detector frame taken within the ψ–*E* grid; (*c*) subtracting the background signal for each point of the ψ–*E* grid; and (*d*) for each energy, averaging the lowest intensities from the different azimuthal positions.

To disentangle the individual contributions to a REXS measurement, we additionally improved the subsequent data analysis with a multilevel routine that takes frames from 2D detectors and was implemented in Python code (Nentwich *et al.*, 2023 ▸), see Fig. 5 ▸. The starting point of our approach is that there is no azimuthal position in a Renninger map that is free of MBD. However, we assume that, for each energy, there are azimuthal values where the influence of MBD is negligible. We now want to identify these automatically and use them to create a clean spectrum. In the following paragraph, we will describe this process and the data treatment involved including different corrections (*e.g.* background, detector, incoming beam intensity…).

The basic idea of the presented approach is that no azimuthal position in a Renninger map exists that is free of MBD. However, a reasonable assumption is that there are azimuthal values where the influence of MBD is negligible (for each energy). We now want to identify these (energy-dependent) azimuthal values automatically and use them to create a clean spectrum. In the following paragraph, we not only describe this process, but also the complete data treatment including different corrections (*e.g.* background, detector, incoming beam intensity *etc.*).

The main steps of this routine are visualized in Fig. 5 ▸. Once the raw data (detector frames and motor positions) are loaded, the frames are normalized to the primary beam and corrected for detector artefacts caused by pixels with different sensitivities (hot and cold pixels) by flatfield correction. In order to separate the background from the actual signal (including MBD) for each of the detector frames, the region containing the diffracted intensity on the detector is marked by the users in step Fig. 5 ▸(*a*). Everything outside this region is considered as constant background for each ψ and *E*, leading to the map shown in Fig. 5 ▸(*b*). The background-corrected signal is obtained by subtracting the average of the intensity readings outside the user-selected region from the average of those values inside. This is done for each azimuth and X-ray energy leading to the map in Fig. 5 ▸(*c*).

In the last step in Fig. 5 ▸(*d*), the Renninger effect is filtered out by evaluating ψ-dependent intensity *I*_*E*_(ψ) for a given X-ray energy *E*. In the case of strong, allowed reflections, both an increase and a decrease of intensity could falsify the measurement. Thus, the median of *I*_*E*_(ψ) is interpreted as the MBD-free intensity. An average cannot be used as extremely high MBD-signal will falsify the results. In the case of weak (*e.g.* ‘forbidden’) reflections, only an increase in intensity due to MBD is observed. In this case, the lowest intensity values are interpreted as MBD-free. For intermediate cases between strong and ‘forbidden’ reflections, the signal can be recovered by individually increasing/decreasing the number of evaluated intensities. In contrast, the background values are reduced to a one-dimensional array over energy by averaging the values corresponding to different ψ values at the same energy.

## 4. Results

**Figure 6:**
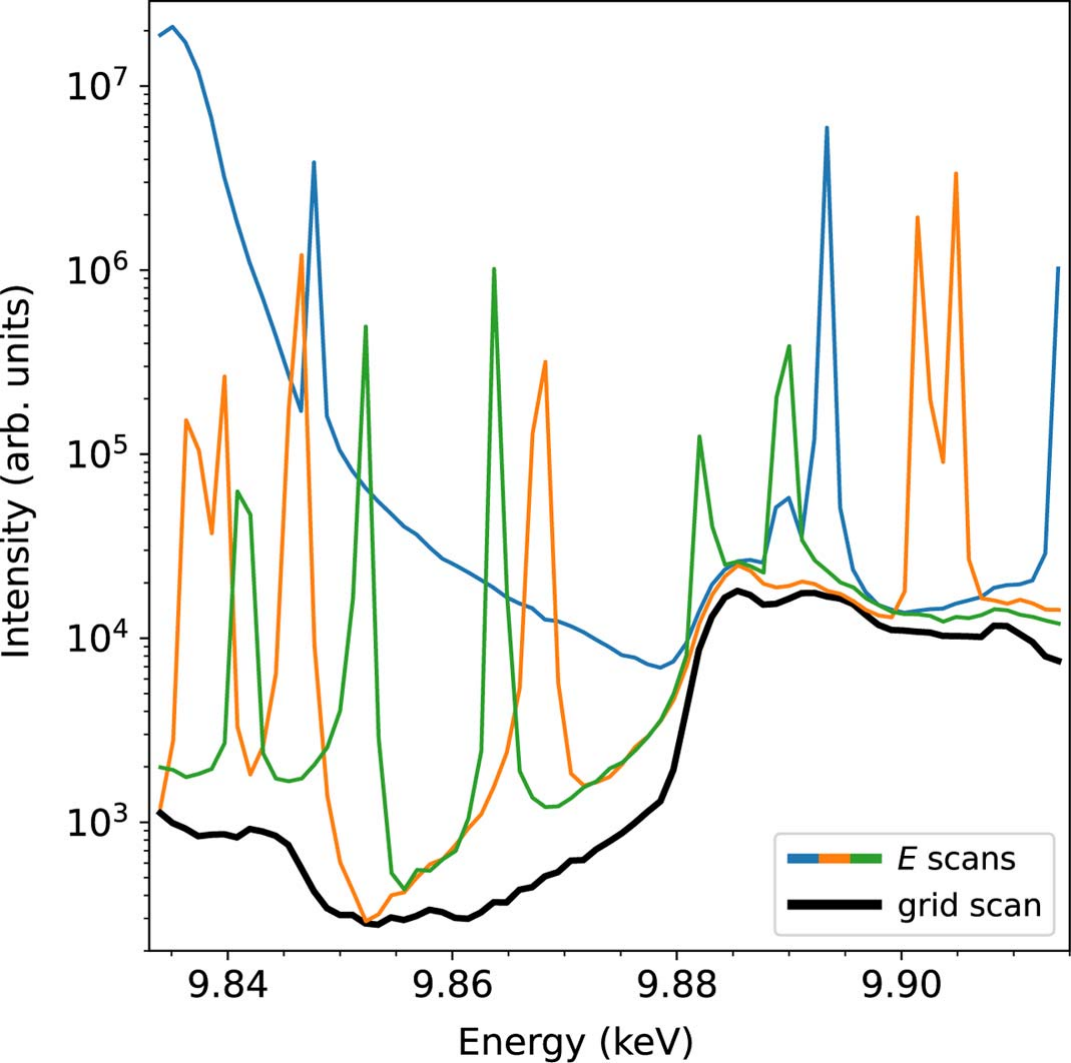
Experimental comparison between fine *E* scans at a few azimuthal values (blue, orange, green; as in Fig. 3 ▸) and ψ–*E* grid scans [black; as in Fig. 5 ▸(*d*)] for the 0 3 15 reflection of congruent LiTaO_3_.

We tested this approach for the ‘forbidden’ 0 3 15 reflection of a congruent LiTaO_3_ single crystal (Crystec, Berlin). The spectra of forbidden reflections are particularly prone to MBD contributions as they are weak. We were able to acquire sufficiently redundant data to extract energy-dependent REX spectra that are almost free from the Renninger effect. Fig. 6 ▸ shows the final result of the procedure using ψ–*E* grid scans [black, as in Fig. 5 ▸(*d*)] in comparison with the initial *E* scans (red, as in Fig. 3 ▸).

**Figure 7:**
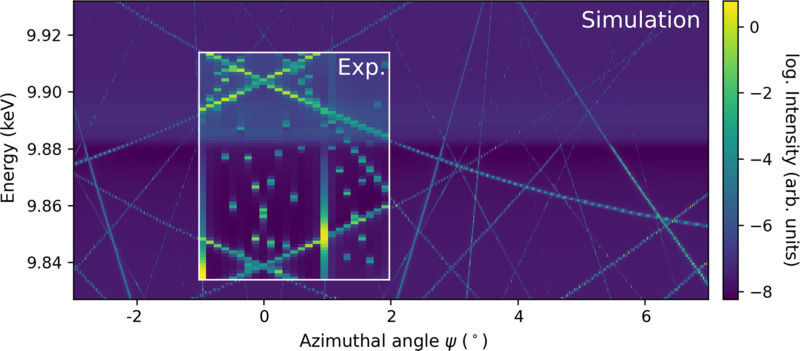
Overlay of the experimental data onto the simulations with *pyasf* and *fdmnes* for the ‘forbidden’ 0 3 15 reflection at the Ta *L*_3_ edge of congruent LiTaO_3_. Both simulations taken individually do not provide the complete information required: *pyasf* (Richter, 2021 ▸) delivers the MBD contribution whereas *fdmnes* (Bunău & Joly, 2009 ▸) delivers the resonant contribution of the ‘forbidden’ reflection.

To benchmark the data-correction procedure (to recover the MBD-free signal), we apply it to a calculated spectrum including MBD, in order to recover the MBD-free signal. The calculations performed for LiTaO_3_ are based on the structure solution of ICSD 9537 (Abrahams & Bernstein, 1967 ▸). The purely energy-dependent part and the MBDs were calculated separately employing *fdmnes* (Bunău & Joly, 2009 ▸) and *pyasf* (Richter, 2021 ▸), respectively, see Fig. 7 ▸. As both programs are not interfaced to each other, the data need to be rescaled to match the experiment. Some discrepancy between calculated and experimental data regarding the MBD-free, ψ-independent part is expected due to an uncertainty in the temperature-induced atomic displacement parameters (ADPs) (Richter *et al.*, 2018 ▸; Weigel *et al.*, 2023 ▸).

**Figure 8:**
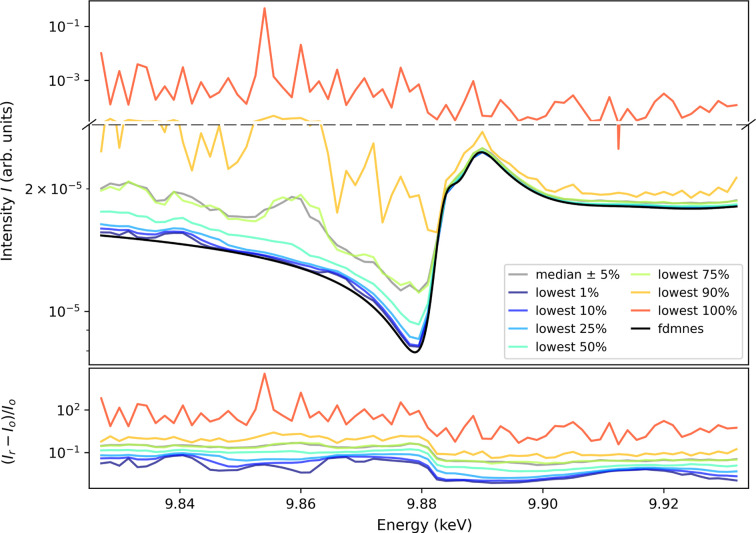
Top: recovery of the MBD-free spectrum based on the synthetic data [step (*d*) in Fig. 5 ▸] for the ‘forbidden’ LiTaO_3_ 0 3 15 reflection, as an example. Different threshold values to discriminate MBD-contribution are chosen. The ‘lowest *i*%’ data are the means of values below the *i*th percentile of intensity *I*_*E*_(ψ) at a given energy. The lowest 100% dataset represents the overall mean. The median (50th percentile) is also shown. Note the different scales of the *y* axis. Bottom: relative differences between the original (*fdmnes*) and the restored signal (*I*_r_ − *I*_o_)/*I*_o_.

Fig. 8 ▸ demonstrates the influence of the choice of the threshold to discriminate the MBD-affected values as described above in step (*d*). We consider again the ‘forbidden’ 0 3 15 reflection of LiTaO_3_. At a given X-ray energy *E*, the threshold is placed at different percentiles (*e.g.* lowest 5%; with the lowest 100% representing the overall mean of the data). Additionally, we present the overall median (equal to the 50th percentile). The bottom part of Fig. 8 ▸ presents the relative differences between the original (*fdmnes*) and the restored signal (*I*_r_ − *I*_o_)/*I*_o_, with their averages ranging between 1.2 × 10^−2^ and 5.7 × 10^2^. As expected, for this weak ‘forbidden’ reflection, the most reasonable choices of threshold for step (*d*) are the lowest percentiles of up to 10%.

Further results are described by Richter *et al.* (2018 ▸) and Weigel *et al.* (2023 ▸), where the present approach was successfully applied to generate clear spectra of allowed reflections and to refine the underlying crystal structure using a fit.

## 5. Conclusions and outlook

We presented an automatable approach to receive clean, Renninger-reflection-free, REX spectra. The approach requires establishing scans of the azimuth angle ψ, *e.g.* as a virtual motor in the beamline control. Subsequently, ψ–*E* grids are measured with a finely screened azimuth angle in the inner loop and moderately stepped energies in the outer one. Employing a Python script, the detector frames corresponding to a ψ–*E* grid are handled and the energy-dependent signal and background are returned, free from the Renninger effect. As a result, clean REX spectra can be measured even at relatively high energies with respect to the unit-cell dimensions (see Fig. 6 ▸) which, in turn, allows us to study anomalous diffraction from crystals at absorption edges located at high energies. Moreover, this facilitates the study of local structure and chemical environments and an extended set of chemical elements in highly perfect single-crystalline materials.

The successful recovery of the REX spectra is mainly limited by the density of the multiple-beam cases: if the density is very low, no recovery is needed as the data are not strongly affected. This is often the case for low energies, small unit cells, and also for weakly scattering samples (*e.g.* powders, thin films) and low-excitation errors. If the density becomes higher, a recovery is possible if still some angular ranges exist that are relatively unaffected from MBD. Otherwise, the presented approach will also fail.

Our approach is especially beneficial for the investigation of ‘forbidden’ reflections, which are particularly sensitive to the Renninger effect. We have demonstrated the effectiveness of the procedure on the energy-dependence of the ‘forbidden’ 0 3 15 reflection of LiTaO_3_ near the Ta *L*_3_ edge.
